# Histological Evidence of Pulmonary Microthrombosis and Vasculitis in Life-Threatening Respiratory Virus Diseases

**DOI:** 10.1093/ofid/ofaa640

**Published:** 2020-12-28

**Authors:** Heather W Dolby, Philippe Potey, Annika B Wilder-Smith, Sara Clohisey, Jonathan E Millar, J Kenneth Baillie, David A Dorward, Christopher D Lucas, Clark D Russell

**Affiliations:** 1 University of Edinburgh Centre for Inflammation Research, Queen’s Medical Research Institute, Edinburgh, United Kingdom; 2 Roslin Institute, Division of Genetics and Genomics, University of Edinburgh, Edinburgh, United Kingdom

**Keywords:** acute respiratory distress syndrome, influenza, SARS coronavirus, thrombosis, vasculitis

## Abstract

Pulmonary microthrombosis and vasculitis occur in fatal coronavirus disease 2019. To determine whether these processes occur in other life-threatening respiratory virus infections, we identified autopsy studies of fatal influenza (n  =  455 patients), severe acute respiratory syndrome ([SARS] n  =  37), Middle East respiratory syndrome (n  =  2), adenovirus (n  =  34), and respiratory syncytial virus (n  =  30). Histological evidence of thrombosis was frequently present in adults with fatal influenza and SARS, with vasculitis also reported.

Thrombotic complications occur with high frequency in coronavirus disease 2019 (COVID-19), involving pulmonary thromboemboli (PTE), deep vein thrombosis, and catheter-related thrombosis, despite thromboprophylaxis with low-molecular-weight heparin (LMWH) [1]. Histologically, pulmonary microthrombi are frequent autopsy findings in fatal COVID-19, even in the absence of macrovascular PTE, and in a subgroup of patients are likely due to immunothrombosis distinct from conventional PTE [2]. This occurs irrespective of receipt of LMWH thromboembolism prophylaxis [2]. There is also mounting evidence of a spectrum of pulmonary vasculitis in COVID-19: neutrophilic capillaritis [3, 4], lymphocytic endotheliitis [5], lymphocyte-plasma cellular arterial vasculitis [6], obliterating endarteritis involving C5aR1^+^ macrophages [7 and MRP8^+^ mononuclear cell vasculitis [2]. These findings, taken together, suggest that pulmonary vasculitis and immunothrombosis could be treatable traits contributing to respiratory failure in subgroups of patients [8]. The established clinical benefit of corticosteroids in COVID-19 supports a causal role for inflammation in severe disease, and trials of anticoagulation are ongoing (ClinicalTrials.gov Identifier NCT04344756 and NCT04406389) [9]. We reviewed human autopsy data from other life-threatening respiratory virus infections to determine whether thrombosis and pulmonary vasculitis occur in other viral infections.

## METHODS

We searched the PubMed (MEDLINE) database using the following keywords: “Adenovirus” OR “Rhinovirus” OR “Metapneumovirus” OR “Parainfluenza” OR “Bocavirus” OR “Influenza” OR “Severe Acute Respiratory Syndrome” OR “Middle East Respiratory Syndrome” OR “Respiratory Syncytial Virus” AND “autopsy” OR “post-mortem” (NOT “COVID-19”). H.W.D. and C.D.R. independently reviewed the search results to select relevant studies. Studies were included that reported pulmonary histopathological findings from postmortem examinations of humans infected with the viruses of interest. We excluded animal studies, studies not written in English, studies potentially reporting overlapping patient cohorts, and studies conducted exclusively in transplant recipients or patients with cancer or immunodeficiencies. For all viruses except respiratory syncytial virus (RSV) and adenovirus, only studies of adult patients were included. Case reports of influenza were excluded, but those of other viruses were included due to the paucity of otherwise available data. Pulmonary histological features were recorded using a standard pro forma (Supplementary Data).

## RESULTS

From 1224 search results, we identified reports for patients with fatal influenza (n = 455 patients; 24 studies), severe acute respiratory syndrome ([SARS] n = 37; 4 studies), Middle East respiratory syndrome ([MERS] n = 2; 2 studies), adenovirus infection (n = 34; 9 studies), and RSV infection (n = 30; 5 studies). The specific histological parameters reported by each study varied, and it was not possible to determine the proportion of patients receiving thromboprophylaxis (Supplementary Data).

### Influenza Virus

Diffuse alveolar damage (DAD), alveolar haemorrhage, and bronchiolitis were the most commonly reported findings in fatal influenza, with neutrophilic bronchopneumonia suggestive of secondary bacterial infection also common (Supplementary Data Table 1). Diffuse alveolar damage was specifically reported in every study and present in 75% of patients. Where data were available, 56% of patients received invasive mechanical ventilation ([IMV]150 of 270). The presence or absence of histological evidence of thrombosis was reported in 14 of 24 studies, with thrombosis present in patients from 12 of these studies and 66 of 317 (21%) patients where quantified. These were most commonly described as fibrin microthrombi. Pulmonary vascular inflammation was less commonly sought by investigators but was present in 5 of 6 studies where it was specified (15 of 79 cases where quantified, 19%). In 4 studies, this was described as perivasculitis and where examined, inflammatory cells involved were CD8^+^ T cells (n = 7) or mononuclear cells (n = 1). One study reported inflammatory infiltrate in the intima of medium vessels and endotheliitis (inflammatory cell type not stated). Vascular involvement in fatal influenza A H1N1 and COVID-19 was compared directly in 1 study [10]. Although prevalence within the cohort was not reported, an infiltrate of CD3^+^ T cells associated with pre- and/or postcapillary walls was described in both diseases. Capillary microthrombi were seen in all cases from both diseases, but they were substantially more prevalent in COVID-19.

### Severe Acute Respiratory Syndrome Coronavirus and Middle East Respiratory Syndrome Coronavirus

Diffuse alveolar damage was present in all fatal cases, and where stated all patients had received IMV. In SARS, thrombosis was reported in 3 of 4 studies, and where quantified it was present in 18 of 26 patients (specified as involving small veins/vessels in 17 of 18 patients). Vasculitis was reported as being present in 2 studies, although its prevalence was not stated (Supplementary Data Table 2). One study described a small vessel pulmonary vasculitis (inflammatory cell type not stated), and another reported vasculitis of small pulmonary veins, involving monocytes, neutrophils, and lymphocytes, with fibrinoid necrosis (in addition to vasculitis of small veins in the heart, liver, kidney, adrenal gland, and muscle, involving monocytes, lymphocytes, and plasma cells). Only 2 case reports of autopsies in fatal MERS were identified, 1 of which reported a CD4^+^ lymphocytic pulmonary artery vasculitis.

### Respiratory Syncytial Virus

There were no reports of pulmonary thrombosis in 4 studies of infants with fatal RSV infection, often in the context of sudden infant death (Supplementary Data Table 3). Vascular inflammation was described in 1 study, with a predominantly mononuclear and occasionally eosinophilic infiltrate surrounding bronchial arteries.

### Adenovirus

Nine studies of fatal adenovirus infection were identified with infrequent reports of thrombosis (2 patients) and vasculitis (2 patients), which was described as necrotising pulmonary vein vasculitis in one case and due to giant cells in the other (Supplementary Data Table 4).

## DISCUSSION

Histological data from human autopsy studies of multiple patient cohorts indicate that pulmonary thrombosis and vasculitis occur in subgroups of adult patients with fatal influenza and SARS. Although thrombosis is likely to be quantitatively greater in COVID-19 [10], it is not unique to this disease. This appears distinct from fatal RSV infection in infants, where thrombosis has not been reported, and adenovirus infection, where there were very infrequent reports. Overall, these histological features do not appear to have been frequently sought systematically by investigators in the past and could be underappreciated features of viral lung injury. In the only study directly comparing influenza A with COVID-19, where thrombosis was specifically sought, it was observed in all influenza cases, although confirmation bias is also a possible explanation for this [10]. Similar appearances have also been described in animal models of influenza and SARS in a variety of species [11–13].

Acute respiratory distress syndrome (ARDS) is the final common pathway of life-threatening viral lung injury. Diffuse alveolar damage is found in open lung biopsy and autopsy studies of approximately 50% of patients with a clinical diagnosis any-cause ARDS (approximately 75% of patients with fatal influenza in this study) [14]. When present, DAD is associated with greater illness severity in ARDS [14]. Pulmonary vascular histological findings are not commonly reported in ARDS autopsy studies but have been reported in 2 studies including 32 patients with a clinical diagnosis of ARDS (nonselected aetiology), with microthrombi present in 28 of 32 [15, 16]. These 2 reports, from 1983 and 1976, would include patients not receiving thromboembolism prophylaxis with heparins, which is now routine. A leucocytoclastic pulmonary vasculitis was reported in one cohort (7 of 22 patients), but only in association with bacterial, viral, or fungal superinfection in patients who died >10 days after intubation [16]. A more recent histological study of ARDS, utilizing open lung biopsy, did not report on the presence of microthrombi or vasculitis, with DAD representing the most common finding, as expected [17].

In COVID-19, circulating markers of thrombosis and endothelial injury (D-dimer, angiopoietin-2, endothelin-1 and von Willebrand Factor A2) increase in a stepwise fashion with disease severity (along the World Health Organization ordinal severity scale) with equivalent concentrations in patients who require IMV and survive compared with patients who die [18]. Similar D-dimer changes occur in patients with H1N1 influenza A infection, which, combined with the increased risk of radiologically diagnosed PTE, supports thrombosis being a relevant process in pathogenesis and not a nonspecific postmortem artifact [19–21].

Biological mechanisms further support the contribution of these processes to pathogenesis. Engulfment of influenza virions by platelets activates TLR7 signaling, leading to prothrombotic neutrophil deoxyribonucleic acid release and aggregation [22]. Platelet degranulation and neutrophil prothrombotic proteomic signatures have been identified in blood from patients with COVID-19 ARDS, and low-density neutrophils from these samples aggregate with platelets [23]. TLR7 is a single-stranded ribonucleic acid (ssRNA) sensor, also involved in the host response to SARS-CoV-2 [24], and activation is likely to occur during infection with other ssRNA viruses. A TLR7/8 agonist upregulates healthy neutrophil Mac-1 platelet binding complex, as seen on neutrophils from COVID-19 ARDS patients [23]. Platelet-endothelial adhesion also occurs in vitro in response to influenza and in people with COVID-19: circulating platelets display a hyperreactive transcriptional response and aggregate with leucocytes [25, 26]. In ARDS, platelets interact with endothelial cells, immune cells, neutrophil extracellular traps, and pathogens, and their activation can lead to immunothrombosis [27].

Evidence from multiple sources supports the role of myeloid recruitment to the lung in COVID-19, which could link inflammation, vasculitis, and immunothrombosis and identify therapeutic targets. In a genome-wide association study of critical illness in COVID-19, a CCR2 variant predicted to increase expression in the lung was identified [28]. Similarly, the chemoattractant C5a and myeloid growth factor granulocyte-macrophage colony-stimulating factor are associated with COVID-19 severity [7, 29]. The C5a-C5aR1 axis has also been associated with H1N1 influenza [30].

Immunothrombosis and vasculitis were identified in cohorts of adult patients, whereas they were infrequent in children. Increased innate immune activation in older adults could contribute to susceptibility to these processes, or alternatively it may relate to clinical differences: most of the children had died suddenly, whereas most adults had received IMV and would have had a more protracted illness [31].

The majority of the identified studies, including COVID-19 and those from the 2009 H1N1 pandemic, have been conducted at a time when critically ill patients routinely receive thromboembolism prophylaxis with LMWH [32]. Thrombosis could therefore be occurring largely independently of the intrinsic pathway in some patients. While we await the results of trials of therapeutic anticoagulation in COVID-19, we suggest that a subgroup of patients with immunothrombosis, especially with vasculitis, may be more responsive to immunomodulatory therapy, distinct from conventional PTE responsive to therapeutic anticoagulation alone. An alternative or potentially complementary approach would be to therapeutically protect the endothelium, as recently discussed in the context of COVID-19 [33]. Such approaches could include administration of nitric oxide, endothelin receptor antagonists, vascular endothelial growth factor antagonists, or other antiproliferative drugs.

## CONCLUSIONS

Overall, we contend that further investigation of the role of immunothrombosis and pulmonary vasculitis in patients with life-threatening respiratory virus infections is warranted, and autopsy studies will have an important role in this. These pathophysiological features could represent treatable traits in subgroups of patients, with implications for prioritizing investigational therapeutic interventions and enriching clinical trials.

## Supplementary Data

Supplementary materials are available at *Open Forum Infectious Diseases* online. Consisting of data provided by the authors to benefit the reader, the posted materials are not copyedited and are the sole responsibility of the authors, so questions or comments should be addressed to the corresponding author.

ofaa640_suppl_Supplementary_Materials
