# Supplementary material for: Histological Evidence of Pulmonary Microthrombosis and Vasculitis in Life-Threatening Respiratory Virus Diseases
Source: Open Forum Infect Dis. 2020 Dec 28;8(2):ofaa640. doi: 10.1093/ofid/ofaa640 (PMC7798721; doi:10.1093/ofid/ofaa640)
Supplement: ofaa640_suppl_Supplementary_Materials [file ofaa640_suppl_supplementary_materials.pdf]

SUPPLEMENTARY DATA

Table 1: Fatal influenza A and B virus infection

Reference	1	2	3	4	5	6	7	8	9	10	11	12	13	14	15	16	17	18	19	20	21	22	23	24
**Cohort details**																								
Patients	100	68	45	44	34	21	20	19	15	12	9	9	8	8	7	6	6	5	4	4	4	3	2	2
Virus	H1N1	H1N1	B	H1N1	H1N1	H1N1	H1N1	H1N1	H1N1	H1N1	A	H1N1	H1N1	H1N1	H1N1	H1N1	H1N1	H1N1	H1N1	H1N1	A	H7N9	H1N1	H5N1
IMV	42	NA	NA	17	12	21	NA	NA	15	NA	4	NA	NA	8	7	6	5	0	NA	4	2	3	2	2
Age (years)	36[b]	27[b]	11[b]	40[b]	42[b]	34[b]	40[b]	57[b]	50[a]	27[a]	53[b]	33[a]	27[b]	40[a]	57[a]	30[a]	39[a]	28[b]	28[a]	36[a]	54[a]	73[a]	41[a]	19[a]
**Lung pathology**																								
DAD	100	42	8	44	25	20	6	9	15	2	7	9	8	8	7	5	5	4	4	4	3	3	2	2
Thrombosis	**17**	**5**	NA	**0**	**9**	**8**	**4**	NA	NA	NA	**1**	**Yes**	**0**	**6**	**7**	NA	**2**	NA	**4**	**3**	NA	NA	NA	NA
Vascular inflammation	NA	NA	NA	**0**	NA	**7**[c]	NA	NA	NA	NA	NA	NA	NA	NA	**Yes**[d]	NA	**1**[e]	NA	**3**[f]	**4**[g]	NA	NA	NA	NA
Bronchopneumonia	29	68	17	2	19	NA	5	1	3	8	NA	Yes	1	6	NA	NA	2	1	3	NA	NA	0	1	0
Bronchiolitis	NA	39	24	19	18	21	NA	13	15	10	8	2	8	NA	NA	2	Yes	NA	4	4	2	NA	0	NA
Alveolar haemorrhage	58	27	30	NA	8	5	13	10	15	3	9	Yes	8	3	NA	6	1	2	NA	3	4	2	1	NA

Table shows numbers unless otherwise stated.

[a]mean; [b]median; [c]perivasculitis involving $CD8^+$ T-cells; [d]perivasculitis involving $CD3^+$ T-cells; [e]perivasculitis involving mononuclear cells; [f]inflammatory infiltrate in medium vessel intima and endotheliitis (cell type not stated); [g]perivasculitis (cell type not stated)

NA: not available; IMV: invasive mechanical ventilation; DAD: diffuse alveolar damage

Table 2: Fatal SARS and MERS coronavirus infection

Reference	25	26	27	28	29	30
**Cohort details**						
Patients	20	8	6	3	1	1
Virus	SARS	SARS	SARS	SARS	MERS	MERS
IMV	NA	NA	6	NA	1	1
Age (years)	68[a]	42[a]	53[a]	48[a]	33	45
**Lung pathology**						
DAD	20	8	6	3	1	1
Thrombosis	**17**	NA	**1**	**Yes**	NA	NA
Vascular inflammation	NA	**Yes**[c]	NA	**Yes**[d]	**1**[e]	NA
Bronchopneumonia	10	0	NA	Yes	NA	NA
Bronchiolitis	NA	0	1	NA	NA	NA
Alveolar haemorrhage	17	NA	NA	NA	1	NA

Table shows numbers unless otherwise stated.

[a]mean; [b]median; [c]small vessel, cell type not stated; [d]small veins, involving monocytes, neutrophils and lymphocytes, with fibrinoid necrosis; [e]$CD4^+$ T-cells

NA: not available; IMV: invasive mechanical ventilation; DAD: diffuse alveolar damage

Table 3: Fatal Respiratory Syncytial Virus infection

Reference	31	32	33	34	35
**Cohort details**					
Patients	12	8	4	4	2
IMV	NA	0	NA	NA	0
Age (months)	NA	3[b]	12[a]	13[b]	19[b]
**Lung pathology**					
DAD	NA	0	1	3	NA
Oedema	12	8	NA	3	NA
Thrombosis	NA	NA	NA	NA	NA
Vascular inflammation	NA	NA	NA	**Yes[c]**	NA
Bronchopneumonia	NA	NA	2	NA	2
Bronchiolitis	7	7	1	4	2
Alveolar haemorrhage	NA	5	NA	1	NA

Table shows numbers unless otherwise stated.
[a]mean; [b]median; [c]mononuclear cells and infrequent eosinophils
NA: not available; IMV: invasive mechanical ventilation; DAD: diffuse alveolar damage

Table 4: Fatal Adenovirus infection

Reference	36	37	38	39	40	41	42	43	44
**Cohort details**									
Patients	18	6	3	2	1	1	1	1	1
IMV	NA	NA	2	0	0	1	1	NA	1
Age	8[a,c]	12.5[a,c]	1[a,c]	5[a,c]	11[a]	2[b]	67[b]	4.7[b]	5[b]
**Lung pathology**									
DAD	18	6	3	NA	1	1	1	NA	1
Oedema	NA	NA	NA	2	1	NA	NA	NA	NA
Thrombosis	NA	NA	**1**	NA	NA	NA	NA	NA	**1**
Vascular inflammation	NA	NA	NA	NA	NA	NA	NA	**1[d]**	**1[e]**
Bronchopneumonia	11	NA	NA	NA	NA	NA	NA	NA	NA
Bronchiolitis	NA	6	3	NA	1	NA	1	NA	NA
Alveolar haemorrhage	NA	NA	3	2	1	1	NA	NA	NA

Table shows numbers unless otherwise stated.
[a]months; [b]years; [c]mean; [d]necrotic vasculitis of pulmonary veins; [e]giant cells
NA: not available; IMV: invasive mechanical ventilation; DAD: diffuse alveolar damage

References

1. Shieh WJ, Blau DM, Denison AM, et al. 2009 pandemic influenza A (H1N1): pathology and pathogenesis of 100 fatal cases in the United States. Am J Pathol **2010**; 177: 166-75.
2. Sheng ZM, Chertow DS, Ambroggio X, et al. Autopsy series of 68 cases dying before and during the 1918 influenza pandemic peak. Proc Natl Acad Sci U S A **2011**; 108: 16416-21.
3. Paddock CD, Liu L, Denison AM, et al. Myocardial Injury and Bacterial Pneumonia Contribute to the Pathogenesis of Fatal Influenza B Virus Infection. The Journal of Infectious Diseases **2012**; 205: 895-905.
4. Shelke VN, Kolhapure RM, Kadam D, et al. Pathologic study of pandemic influenza A (H1N1) 2009 cases from India. Pathol Int **2012**; 62: 36-42.
5. Gill JR, Sheng ZM, Ely SF, et al. Pulmonary pathologic findings of fatal 2009 pandemic influenza A/H1N1 viral infections. Arch Pathol Lab Med **2010**; 134: 235-43.
6. Mauad T, Hajjar LA, Callegari GD, et al. Lung pathology in fatal novel human influenza A (H1N1) infection. Am J Respir Crit Care Med **2010**; 181: 72-9.
7. Nakajima N, Sato Y, Katano H, et al. Histopathological and immunohistochemical findings of 20 autopsy cases with 2009 H1N1 virus infection. Modern Pathology **2012**; 25: 1-13.
8. Tamme K, Minajeva A, Adamson V, Ristmägi K, Põder J, Lutsar I. Clinical and pathological findings of fatal 2009-2010 pandemic influenza A (H1N1) infection in Estonia. Medicina (Kaunas) **2012**; 48: 48-56.
9. Buttignol M, Pires-Neto RC, Rossi e Silva RC, Albino MB, Dolhnikoff M, Mauad T. Airway and parenchyma immune cells in influenza A(H1N1)pdm09 viral and non-viral diffuse alveolar damage. Respiratory Research **2017**; 18: 147.
10. Brooks EG, Bryce CH, Avery C, Smelser C, Thompson D, Nolte KB. 2009 H1N1 fatalities: the New Mexico experience. J Forensic Sci **2012**; 57: 1512-8.
11. Feldman PS, Cohan MA, Hierholzer WJ, Jr. Fatal Hong Kong influenza: a clinical, microbiological and pathological analysis of nine cases. Yale J Biol Med **1972**; 45: 49-63.
12. Bal A, Suri V, Mishra B, et al. Pathology and virology findings in cases of fatal influenza A H1N1 virus infection in 2009-2010. Histopathology **2012**; 60: 326-35.
13. Rosen DG, Lopez AE, Anzalone ML, et al. Postmortem findings in eight cases of influenza A/H1N1. Modern Pathology **2010**; 23: 1449-57.
14. Harms PW, Schmidt LA, Smith LB, et al. Autopsy findings in eight patients with fatal H1N1 influenza. Am J Clin Pathol **2010**; 134: 27-35.

15. Ackermann M, Verleden SE, Kuehnel M, et al. Pulmonary Vascular Endothelialitis, Thrombosis, and Angiogenesis in Covid-19. N Engl J Med **2020**; 383: 120-8.
16. Nin N, Sánchez-Rodríguez C, Ver LS, et al. Lung histopathological findings in fatal pandemic influenza A (H1N1). Med Intensiva **2012**; 36: 24-31.
17. Calore EE, Uip DE, Perez NM. Pathology of the swine-origin influenza A (H1N1) flu. Pathology - Research and Practice **2011**; 207: 86-90.
18. Carmona F, Carlotti APCP, Ramalho LNZ, Costa RS, Ramalho FS. Evidence of Renal Infection in Fatal Cases of 2009 Pandemic Influenza A (H1N1). American Journal of Clinical Pathology **2011**; 136: 416-23.
19. Soto-Abraham MV, Soriano-Rosas J, Díaz-Quiñónez A, et al. Pathological changes associated with the 2009 H1N1 virus. N Engl J Med **2009**; 361: 2001-3.
20. Marchiori R, Bredt CSdO, Campos MMFd, Negretti F, Duarte PAD. Pandemic influenza A/H1N1: comparative analysis of microscopic lung histopathological findings. einstein (São Paulo) **2012**; 10: 306-11.
21. Fujita J, Ohtsuki Y, Higa H, et al. Clinicopathological findings of four cases of pure influenza virus A pneumonia. Intern Med **2014**; 53: 1333-42.
22. Yu L, Wang Z, Chen Y, et al. Clinical, Virological, and Histopathological Manifestations of Fatal Human Infections by Avian Influenza A(H7N9) Virus. Clinical Infectious Diseases **2013**; 57: 1449-57.
23. Mukhopadhyay S, Philip AT, Stoppacher R. Pathologic Findings in Novel Influenza A (H1N1) Virus (“Swine Flu”) Infection: Contrasting Clinical Manifestations and Lung Pathology in Two Fatal Cases. American Journal of Clinical Pathology **2010**; 133: 380-7.
24. To KF, Chan PK, Chan KF, et al. Pathology of fatal human infection associated with avian influenza A H5N1 virus. J Med Virol **2001**; 63: 242-6.
25. Hwang DM, Chamberlain DW, Poutanen SM, Low DE, Asa SL, Butany J. Pulmonary pathology of severe acute respiratory syndrome in Toronto. Mod Pathol **2005**; 18: 1-10.
26. Gu J, Gong E, Zhang B, et al. Multiple organ infection and the pathogenesis of SARS. J Exp Med **2005**; 202: 415-24.
27. Nicholls JM, Poon LL, Lee KC, et al. Lung pathology of fatal severe acute respiratory syndrome. Lancet **2003**; 361: 1773-8.
28. Ding Y, Wang H, Shen H, et al. The clinical pathology of severe acute respiratory syndrome (SARS): a report from China. J Pathol **2003**; 200: 282-9.
29. Alsaad KO, Hajeer AH, Al Balwi M, et al. Histopathology of Middle East respiratory syndrome coronovirus (MERS-CoV) infection - clinicopathological and ultrastructural study. Histopathology **2018**; 72: 516-24.
30. Ng DL, Al Hosani F, Keating MK, et al. Clinicopathologic, Immunohistochemical, and Ultrastructural Findings of a Fatal Case of Middle East Respiratory Syndrome Coronavirus Infection in the United Arab Emirates, April 2014. Am J Pathol **2016**; 186: 652-8.
31. Daş T, Sargan A, Yağmur G, et al. Viral Pneumonias in Forensic Autopsies: Evaluation and Classification of Histopathologic Changes With Microbiologic Correlation. Am J Forensic Med Pathol **2016**; 37: 255-63.
32. Ikeda T, Tani N, Aoki Y, Shida A, Morioka F, Ishikawa T. Immunohistochemical detection of respiratory syncytial virus at autopsy. Pediatr Int **2019**; 61: 781-91.
33. Weber MA, Hartley JC, Ashworth MT, Malone M, Sebire NJ. Virological investigations in sudden unexpected deaths in infancy (SUDI). Forensic Sci Med Pathol **2010**; 6: 261-7.
34. Johnson JE, Gonzales RA, Olson SJ, Wright PF, Graham BS. The histopathology of fatal untreated human respiratory syncytial virus infection. Modern Pathology **2007**; 20: 108-19.
35. Kakimoto Y, Seto Y, Ochiai E, Satoh F, Osawa M. Cytokine Elevation in Sudden Death With Respiratory Syncytial Virus: A Case Report of 2 Children. Pediatrics **2016**: e20161293.
36. Siminovich M, Murtagh P. Acute lower respiratory tract infections by adenovirus in children: histopathologic findings in 18 fatal cases. Pediatr Dev Pathol **2011**; 14: 214-7.
37. Garcia AGP, Fonseca MEF, De Bonis M, Ramos HIB, Ferro ZPA, Nascimento JP. Morphological and Virological Studies in Six Autopsies of Children with Adenovirus Pneumonia. Mem Inst Oswaldo Cruz **1993**; 88: 141-7.
38. Kim JS, Han HS, Park SH, Chun YK, Lee HJ, Chi JG. Neonatal Adenoviral Pneumonia Report of Three Autopsy Cases. JKMS **1997**; 12: 146-50.
39. Quan L, Zhu B, Ishida K, Taniguchi M, Oritani S, Kamikodai Y, Fujita MQ, Maeda H. Sudden infant death attributed to peracute pulmonary infection: two autopsy cases. Leg Med (Tokyo) **2000**; 2: 79-83.
40. Henson D, Mufson MA. Myocarditis and pneumonitis with type 21 adenovirus infection. Association with fatal myocarditis and pneumonitis. Am J Dis Child **1971**; 121:334-6.
41. Kim KS, Gohd RS. Fatal pneumonia caused by adenovirus type 35. Am J Dis Child **1981**; 135: 473-5.
42. Castelli JB, Siciliano RF, Vieira RD, Aiello VD, Strabelli TM. Fatal adenoviral necrotizing bronchiolitis case in a post-cardiac surgery intensive care unit. Braz J Infect Dis **2011**;15: 285-7.
43. Hoagland MH, Zinkham WH, Hutchins GM. Generalized venocentric lesions in the virus-associated hemophagocytic syndrome. Hum Pathol **1986**; 17: 195-8.
44. Vaideeswar P, Bavdekar SB, Jadhav SM, Balan R, Pandit SP. Necrotizing adenoviral pneumonia: manifestation of nosocomial infection in pediatric intensive care unit. Indian J Pediatr **2008**; 75: 1171-4.
